# Concise review: programming human pluripotent stem cells into blood

**DOI:** 10.1111/bjh.14010

**Published:** 2016-03-21

**Authors:** Jennifer Easterbrook, Antonella Fidanza, Lesley M. Forrester

**Affiliations:** ^1^ MRC Centre for Regenerative MedicineUniversity of EdinburghEdinburghUK

**Keywords:** pluripotent stem cells, differentiation, hematopoietic stem cells, hematopoietic progenitors cells, transcription factors, programming

## Abstract

Blood disorders are treated with cell therapies including haematopoietic stem cell (HSC) transplantation as well as platelet and red blood cell transfusions. However the source of cells is entirely dependent on donors, procedures are susceptible to transfusion‐transmitted infections and serious complications can arise in recipients due to immunological incompatibility. These problems could be alleviated if it was possible to produce haematopoietic cells *in vitro* from an autologous and renewable cell source. The production of haematopoietic cells in the laboratory from human induced pluripotent stem cells (iPSCs) may provide a route to realize this goal but it has proven challenging to generate long‐term reconstituting HSCs. To date, the optimization of differentiation protocols has mostly relied on the manipulation of extrinsic signals to mimic the *in vivo* environment. We review studies that have taken an alternative approach to modulate intrinsic signals by enforced expression of transcription factors. Single and combinations of multiple transcription factors have been used in a variety of contexts to enhance the production of haematopoietic cells from human pluripotent stem cells. This programming approach, together with the recent advances in the production and use of synthetic transcription factors, holds great promise for the production of fully functional HSCs in the future.

## The quest for engraftable HSCs

The generation of an unlimited supply of clinical‐grade, engraftable haematopoietic stem cells (HSCs) and fully functional mature blood cells is a highly sought after goal for clinical haematologists. HSC transplantation is currently the most widely used regenerative therapy in clinical practice, used as a potentially curative treatment for a wide range of malignant and non‐malignant conditions. The current major source of HSCs is from peripheral blood following mobilization from bone marrow (BM), with BM itself and umbilical cord blood (UCB) providing alternative sources. However, problems with all of these sources include a significant shortage of appropriate donor supplies, the requirement for immune compatibility and risk of transmission of infectious or malignant disease. The ultimate goal of this field is therefore to produce a reliable and scalable source of HSCs capable of efficient and complete long‐term engraftment. In addition to their direct therapeutic application for BM transplantation, HSCs could provide a source of mature haematopoietic cells for other therapeutic purposes such as red blood cell and platelet transfusions and for drug testing and modelling of both human development and haematological malignancies.

Pluripotent stem cells (PSCs) could potentially provide the answer to this quest (Table 1). These cells are capable of extensive self‐renewal in the laboratory and can be differentiated into any cell type of the body, including blood cells. One type of PSC, known as human embryonic stem cells (hESCs) is derived from the inner cell mass of the embryonic blastocyst but, although they provide an excellent tool to study human biology, ethical concerns associated with their origin limit their use in the clinic. Hence the advent of induced‐pluripotent stem cells (iPSCs) led to great optimism in the field as a potentially limitless source of clinical‐grade, immunologically‐matched hematopoietic cells (Kaufman, 2009). Human iPSCs can be produced by reprogramming mature adult cells, such as skin cells, into stem cells with the ‘pluripotency‐associated transcription factor’‐encoding genes, *POU5F1, KLF4, SOX2* and *MYC* (Takahashi *et al*, 2007). Their extensive self‐renewal capacity and ability to differentiate into any cell type means that they can provide an autologous source for any cell type.

**Table 1:** Key definitions

PSC	Pluripotent stem cell	Includes both ESCs and iPSCs
iPSC	Induced pluripotent stem cell	Derived from reprogrammed adult somatic cells
hESC	Human embryonic stem cell	Derived from the inner cell mass of a blastocyst
EB	Embryoid body	Three dimensional aggregate of PSCs in suspension
HSC	Haematopoietic stem cell	Capacity for long‐term multilineage engraftment & serial transplantation
HPC	Haematopoietic progenitor cell	Precursor cell lacking true HSC properties above
TF	Transcription factor	Protein controlling DNA transcription
HE	Haemogenic endothelium	Specialized endothelium with haematopoietic potential

Attempts to generate haematopoietic cell types from iPSCs and hESCs have thus far involved differentiation protocols that include the step‐wise addition of cytokines in serum‐free conditions, differentiation of PSCs in three‐dimensional structures known as embryoid bodies (EBs) or co‐culture on stromal cells (Kaufman *et al*, 2001; Zambidis *et al*, 2005; Kennedy *et al*, 2007; Ledran *et al*, 2008; Salvagiotto *et al*, 2011). There has been some success in producing multilineage progenitors and cells that are capable of limited *in vivo* reconstitution but there are no robust, reproducible protocols that can generate long‐term reconstituting HSCs. While mature blood cells, such as macrophages (Choi *et al*, 2011), dendritic cells, and erythrocytes (Kobari *et al*, 2012), can be produced, these protocols also have significant limitations. For instance, hESC‐derived erythroid cells predominantly express fetal rather than adult globins and fail to enucleate efficiently (Mountford *et al*, 2010; Mazurier *et al*, 2011). It is noteworthy that many of these studies report significant variations in the haematopoietic potential of different hESC/iPSC lines in identical culture conditions suggesting that there are specific intrinsic signals that regulate the production of haematopoietic cells *in vitro* (Melichar *et al*, 2011). Could manipulation of these intrinsic signals be the solution? While it is clear that intrinsic signalling involves highly complex genetic networks, specific master regulatory transcription factors (TFs) have been identified which regulate haematopoietic development (Lessard *et al*, 2004; Teitell & Mikkola, 2006; Wilson *et al*, 2011). The production of HSCs from human PSCs *in vitro* could be enhanced potentially by manipulating the expression of these key TFs.

## Genetic programming with transcription factors

**Figure 1:**
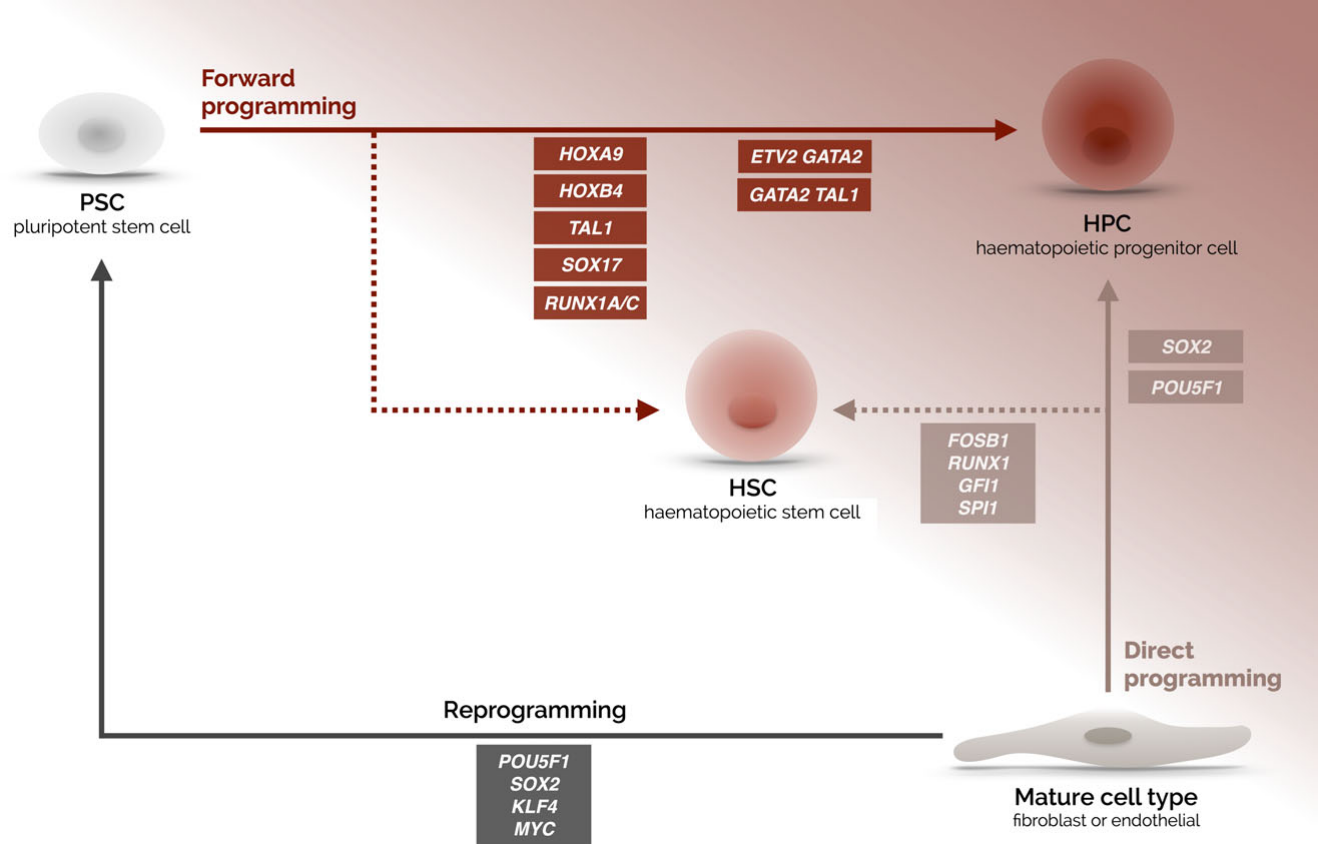
Programming strategies employed for the production of human haematopoietic cells *in vitro*. The production of haematopoietic progenitors cells (HPCs) from human pluripotent stem cells (PSCs) has been enhanced by the overexpression of single or multiple TFs (forward programming). TFs have also been used to programme other mature cell types, such as endothelial cells and fibroblasts, into HPCs (direct programming). The production of multilineage cells with serial transplantation capacity has been achieved *in vitro* (dotted arrow) but these cells lacked T cell potential *in vivo* so it is premature to consider these as fully functional haematopoietic stem cells (HSCs). Classic reprogramming of fibroblasts into PSCs with the four TFs reported by Takahashi *et al*., (2007) has been included for completeness. See text for references.

The concept of TF programming, whereby overexpression of master TFs in one cell type can convert the cell into a different functional cell, was first demonstrated by the conversion of murine fibroblasts to cells with myogenic properties with the single TF‐encoding gene, *Myod1* (Davis *et al*, 1987). However, arguably the most acclaimed example was the landmark report that fully differentiated somatic cells can be reprogrammed to PSCs by the exogenous expression of just four TFs (Takahashi & Yamanaka, 2006). TF programming has also been used to produce haematopoietic progenitor cells (HPCs)/HSCs either from a mature differentiated cell type (herein referred to as direct programming) or from PSCs (herein referred to as forward programming) (Fig 1). Human dermal fibroblasts were converted to myeloid‐restricted multilineage HPCs in a direct programming strategy by the ectopic expression of the pluripotency‐associated TFs, *POU5F1* (Szabo *et al*, 2010) and *SOX2* (Pulecio *et al*, 2014). Similarly, two studies demonstrated that murine fibroblasts could be directly converted to HPCs using combinations of haematopoietic TFs (Pereira *et al*, 2013; Batta *et al*, 2014). These direct programming strategies will not be discussed in detail here as an excellent review on this topic has been published recently (Ebina & Rossi, 2015). However, it is clear that this direct programming strategy has not been able to generate robust long‐term repopulating HSCs with multilineage potential.

One approach to this problem has been to use a starting cell that has a more similar epigenetic memory to functional haematopoietic cells. For example, direct programming of human umbilical vein and dermal microvascular endothelial cells with *FOSB, GFI1, RUNX1* and *SPI1* resulted in the production of multipotent HPCs capable of long‐term engraftment and serial transplantation. However, although a small but significant population of T cells were generated *in vitro* when the expression of *SPI1* was temporally restricted, T cells were not detected in engrafted recipients (Sandler *et al*, 2014). This study emphasized the importance of the microenvironment as reprogrammed cells had to be propagated within an instructive vascular niche to induce the outgrowth of multipotent HPCs.

Transient expression of *Runx1, Hlf, Lmo2, Prdm5, Pbx1,* and *Zfp37* in primary murine lymphoid and myeloid progenitors further supported the hypothesis that reducing the epigenetic barrier could provide a route to the successful production of HSCs (Riddell *et al*, 2014). Cells with clonal multilineage differentiation potential [termed induced‐HSCs (i‐HSCs)] capable of serial transplantation were generated using this strategy. However, although the TFs were delivered to their target cells *ex vivo*, the cells were then returned to the haematopoietic inductive microenvironment *in vivo*, again highlighting the fact that the environment plays an essential role in maturation and maintenance of reprogrammed cells. While an exciting advance, this study required HSCs to be selected and expanded *in vivo* and clearly this type of strategy would be difficult to replicate routinely in the clinical setting.

## Forward programming of hPSCs

While different starting cells have been employed in an attempt to ‘lower the epigenetic barrier’, human PSCs arguably provide the best genomic landscape for successful production of HSCs. Forward programming of human PSCs with appropriate TFs is therefore an exciting prospect (Fig 1). In this review we highlight recent studies that have used forward programming in both human ESCs and iPSCs to enhance production of HPCs. We summarize the role of the chosen TFs in haematopoiesis *in vivo*, describe the selection strategies & differentiation methods employed and discuss the reported effects on HPC/HSC production (Table 2). A number of strategies have used single TFs to enhance haematopoietic differentiation but more recently combinatorial strategies involving multiple TFs have been used.

**Table 2:** Effect of transcription factor overexpression on production of HPCs and HSCs from human PSCs

Reference	Genes	How gene identified	Overexpression system	Differentiation System	Cell Lines	HPC production (CFU‐C)	bHSC production
Yung *et al* (2011)	*TAL1*	Transcriptional profiling of hESC‐derived CD31^+^KDR^+^ cells and literature	Lentivirus	EB (Serum‐free Feeder‐free)	hESCs: H1, H9	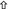 CFU‐Cs (mostly CFU‐E/BFU‐E)	Nil
Real *et al* (2012)	*TAL1*	Literature	Lentivirus	EB and OP9 co‐culture	hESCs: H9, AND1, HS181	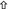 CFU‐Cs	Nil
Ran *et al* (2013)	*RUNX1A*	Literature	Lentivirus	Spin EB	hESCs: H9 hiPSCs: BC1, iCB5	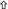 CFU‐Cs	Short‐term engraftment
Real *et al* (2013)	*RUNX1C*	Literature	Lentivirus	EB and OP9 co‐culture	hESCs: H9, AND1, HS181	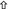 CFU‐Cs	Nil
Nakajima‐Takagi *et al* (2013)	*SOX17*	*In vitro* screen of 13 haematopoietic regulators by overexpression in hPSC‐derived CD34^+^CD43^−^ cells	Retrovirus	EB and OP9 co‐culture	hESCs: H1 hiPSCs: TkCBV4‐7	ND ( 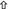 CD34^+^ CD43^+^ CD45^−/low^ cells)	ND
Ramos‐mejia *et al* (2014)	*HOXA9*	Previous work: Differential expression in cord blood CD34^+^ vs hESC‐derived CD34^+^	Lentivirus	EB and OP9 co‐culture	hESCs: H9, AND1	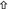 CFU‐Cs (skewed to CFU‐G)	Nil
Forrester and Jackson (2012)	*HOXB4*	See review	Various	Various	Various	Variable	Nil
Elcheva *et al* (2014)	*GATA2/ETV2* *GATA2/TAL1*	*In vitro* screen of 27 candidate genes using over‐expression in hPSCs	Lentivirus and modified mRNA	OP9 co‐culture	hESCs: H1, H9 hiPSCs: DF‐19‐9‐7T, DF‐4‐3‐7T	GATA2/ETV2: pan‐myeloid GATA2/TAL1: erythro‐megakaryocytic	Nil
Doulatov *et al* (2013)	*HOXA9, ERG, RORA, SOX4, MYB*	Compared expression in HSCs vs progenitors/mature cells. *In vitro*/*in vivo* screens using overexpression system	Lentivirus *(Constitutive & Inducible)*	EB	ahESCs: CHB6 ahiPSCs: MSC‐IPS1	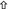 CFU‐GEMM	Short term myeloid & erythroid engraftment

ND, Not determined; EB, Embryoid body; hESCs, human embryonic stem cells; hiPSCs, human induced‐pluripotent stem cells; CFU‐C, Colony‐forming unit in culture; CFU‐E, CFU‐Erythroid; CFU‐G, CFU‐Granulocyte; CFU‐GEMM, CFU‐granulocyte/erythroid/monocyte/megakaryocyte; BFU‐E, Blast‐forming unit in culture‐erythroid; HSC, Haematopoietic stem cell; HPC, Haematopoietic progenitor cell.

a With the exception of Doulatov *et al* (2013) who used hPSC‐derived CD34^+^CD45^+^ as starting cells, these are all examples of forward programming of human PSCs. All overexpression systems are constitutive unless specified otherwise.

b ’HSC production’ denotes *in vivo* engraftment.

## Forward programming of human PSCs with single transcription factors

*TAL1* (T cell acute lymphocytic leukaemia 1), also known as *SCL* (stem cell leukaemia), is a critical haematopoietic regulator that plays a key role in both embryonic and adult HSC specification and its rearrangement is associated with several human leukaemias (Lécuyer & Hoang, 2004). Mice carrying a homozygous deletion of the *Tal1* gene do not survive beyond embryonic day 9·5 due to a failure of haematopoietic development (Shivdasani *et al*, 1995) but its precise role remains unclear. In one study, *Tal1* was shown to be indispensable for the *establishment* of the haemogenic endothelium (HE) (Lancrin *et al*, 2009), but in another it is allegedly dispensable for the development of the haemangioblast but essential for subsequent haematopoietic *commitment* (D'Souza *et al*, 2005).

The role of *TAL1* in early human haematopoiesis has been investigated using the hESC differentiation system (Yung *et al*, 2011; Real *et al*, 2012). *TAL1* was identified as being the most highly upregulated transcript in the emerging haemangioblast (CD31^+^CD309^+^ cells) during the first 4 days of a feeder‐free, serum‐free hESC haematopoietic differentiation protocol (Yung *et al*, 2011). Subsequent experiments, in which the *TAL1* cDNA was overexpressed more than 100‐fold in hESCs, demonstrated enhanced differentiation of meso‐endodermal lineages and increased differentiation to all myeloid lineages. *TAL1* overexpression also accelerated formation of erythro‐megakaryocytic progenitors and most notably accelerated erythroid differentiation. Intra‐splenic transplantation of *TAL1*‐overexpressing hESC‐derived haematopoietic cells enhanced recovery of immunocompromised mice from induced acute haemolytic anemia but no significant engraftment of cells was detected. The authors suggested that the observation could indicate that overexpression of *TAL1* might also have a paracrine effect comparable to that reported for *HOXB4* (Jackson *et al*, 2012) but this clearly requires further investigation. *TAL1* was also reported to be expressed in hESCs‐derived haemato‐endothelial progenitors (CD45^‐^CD31^+^CD34^+^) and in CD45^+^ cells using an OP9 co‐culture differentiation system (Real *et al*, 2012). Overexpression of *TAL1* in that system accelerated the production of the haemato‐endothelial progenitors and the subsequent differentiation into HPCs (CD34^+^CD45^+^) with significant clonogenic potential, but these cells failed to engraft *in vivo*. Silencing of endogenous *TAL1* using sh*TAL1* abrogated haematopoietic specification of hESCs, which further supports its key role in that process. Thus, in contrast to the conflicting findings in the murine system, studies in the hESC system suggest that *TAL1* is essential *both* for the establishment of the HE and its subsequent haematopoietic commitment and is a good candidate for forward programming strategies.

*RUNX1 (AML1, CBFA2, PEBP2aB)* is essential for the establishment of the definitive haematopoietic system during development and is a key transcriptional regulator of normal and malignant haematopoiesis (Chen *et al*, 2009; Lam & Zhang, 2012; Liakhovitskaia *et al*, 2014). While there are at least 12 different mRNA isoforms, three main protein isoforms (RUNX1A/B/C) are most well studied. Overexpression of *RUNX1A* using constitutive lentiviral transduction in a defined spin EB differentiation system significantly enhanced haematopoietic differentiation of human PSCs (Ran *et al*, 2013). Gene expression analysis revealed that *RUNX1A* forced lineage commitment to mesoderm and specifically enhanced haemogenic differentiation. There was expansion of HPCs and establishment of ‘definitive HSCs’ as evidenced by higher β globin expression and multilineage *in vivo* engraftment of immunodeficient (NSG) mice at 9 weeks. However, while these *in vivo* engraftment results were extremely promising, there remains the possibility that the engraftment could have been due to *RUNX1A*‐mediated transformation of hESC‐derived cells, particularly given it is known to contribute to leukaemogenesis (Real *et al*, 2013). In these experiments *RUNX1A* was expressed at very high, non‐physiological levels (>700‐fold higher than normal) and the *RUNX1A*‐expressing CD45^+^CD34^+^ cells showed a surprisingly greater rate of expansion (>25‐fold) compared to their CD34^+^ UCB‐derived counterparts. *RUNX1A*‐expressing cells were limited in their terminal differentiation capacity with approximately 80% of the CD45^+^ cells retaining the CD34 marker, indicating that they retained a progenitor‐like phenotype. Furthermore, although 100% of the analysed mice showed some level of engraftment, multilineage analysis revealed that only a relatively small proportion of the engrafted cells expressed mature haematopoietic cell markers (~11% myeloid, 8% lymphoid, 5% erythroid), indicating that over 70% of the repopulation consisted of undifferentiated progenitors.

In another study using an OP9 co‐culture differentiation system, the emergence of haematopoietic cells was shown to parallel more closely to the expression of the *RUNX1C*, rather than the *RUNX1A* isoform but while overexpression of *RUNX1C* also accelerated and enhanced the production of haemato‐endothelial cells, no *in vivo* engraftment was detected (Real *et al*, 2013). Hence, although *RUNX1A* overexpression holds promise as a potential forward programming factor for *in vitro* HSC production, further studies are required to investigate its possible transformation potential. This issue could be addressed using an inducible rather than constitutive expression system or by using cell‐permeable proteins rather than lentiviral transduction of cDNA sequences. Exemplified by the use of HOXB4‐TAT proteins (Krosl *et al*, 2003), such cell‐permeable TFs would have a limited half‐life and oncogenic transformation and/or insertional mutagenesis associated with genomic integration would be avoided.

*SOX17* (Sry box 17) plays a role in a number of developmental processes, including endoderm (Hudson *et al*, 1997; Kanai‐Azuma *et al*, 2002) and vascular development (Matsui *et al*, 2006)^,^ and has been shown to be important for the regulation of murine fetal and neonatal, but not adult, HSCs. Overexpression of *SOX17* has been reported to confer fetal characteristics onto adult HPCs (He *et al*, 2011). To identify genes promoting haematopoietic development of hPSCs, genes encoding 13 TFs known to be haematopoietic regulators (including *RUNX1, GATA2, HOXB4, TAL1, SOX17*) were overexpressed in hESC/iPSC‐derived CD34^+^CD43^−^ endothelial cells (Nakajima‐Takagi *et al*, 2013)*. SOX17* was found to be the only TF that promoted cell growth and supported the expansion of CD34^+^43^+^45^−/low^ cells expressing the HE marker, CD144 (VE‐Cadherin). *SOX17* was expressed at higher levels in CD34^+^43^−^ cells compared to CD34^+^43^+^45^−^ (‘pre‐HPCs’) and CD34^+^43^+^45^+^ (‘HPCs’). Overexpression of *SOX17* promoted expansion of HE‐like cells but inhibited haematopoietic differentiation of pre‐HPCs and HPCs and reprogrammed them into HE‐like cells, while depletion of *SOX17* in pre‐HPCs did not affect their haematopoietic differentiation. Hence, this study suggests that *SOX17* is a master regulator of HE but must be downregulated thereafter to allow haematopoietic differentiation to occur. The use of *SOX17* in a forward programming strategy to generate haematopoietic cells from iPSCs will therefore require an expression strategy that will allow it to be switched on and off at the appropriate time during the differentiation protocol.

HOXB4 belongs to the homeobox‐containing class of proteins that are well known to play an important role in haematopoietic development (Alharbi *et al*, 2013). Enforced expression of *HOXB4* results in the expansion of adult HSCs *ex vivo* (Antonchuk *et al*, 2002; Schiedlmeier *et al*, 2003) and has been shown to confer long‐term reconstitution ability on primitive yolk sac cells and mouse ESCs (Kyba *et al*, 2002). It is widely reported to enhance the production of haematopoietic cells from murine ESCs and its activity appears to be mediated both via cell autonomous & paracrine mechanisms (Jackson *et al*, 2012). However, its effect on the differentiation of hESCs is less clear. Variable results in hESC studies are likely to be due to the different strategies used to express HOXB4 protein resulting in differing levels of expression and the variety of differentiation systems employed (Forrester & Jackson, 2012). Using a well‐defined serum‐free, feeder‐free hPSC differentiation system and an inducible expression strategy, our group has recently shown that activation of an inducible HOXB4‐ER^T2^ fusion protein enhanced the production of multipotential progenitors, it has no effect on subsequent erythroid maturation and hence failed to produce a more ‘definitive’ phenotype (Jackson *et al*., 2016).

HOXA9 is another homeobox‐containing protein that plays a crucial role in haematopoiesis *in vivo*. It is expressed in HPCs and is downregulated upon differentiation, knockout mice display significant haematopoietic defects and it is frequently overexpressed in human leukaemias (Alharbi *et al*, 2013). In a study to compare the expression profiles of hESC‐derived and cord blood‐derived HPCs, *HOXA9* was found be to expressed at a significantly lower level in hESC‐derived HPCs and so was considered as one of the factors that could be used to improve the function of hESC‐derived cells (Ramos‐mejia *et al*, 2014). Using a lentiviral expression system this study demonstrated that enforced expression of *HOXA9* in hESCs promoted the commitment of haemogenic precursors (CD31^+^CD34^+^CD45^−^) to clonogenic HPCs and mature CD45^+^ cells. However, *HOXA9* alone was not sufficient to confer *in vivo* long‐term engraftment potential to hESC‐derived HPCs, reinforcing the notion that multiple TFs will be required for the production of definitive HSCs from human PSCs.

Through ectopic expression of TFs in various haematopoietic differentiation systems, the studies reviewed here have affirmed their key role in haematopoiesis and have revealed important insights into the process of human haematopoietic development. Importantly however, apart from the potentially promising results with *RUNX1A*, the overexpression of single TFs has so far failed to produce *in vivo* engraftable HSCs. Given the complex nature of haematopoietic commitment, it is perhaps not surprising that a single TF alone could not initiate the entire haematopoietic programme.

## Forward programming of human PSCs with multiple transcription factors

The concomitant overexpression of multiple TFs was employed to elucidate the transcriptional control of HE production. For this, 27 candidate genes were screened that were known from the literature to be key transcriptional regulators of both mesodermal and angio‐haematopoietic specification as well as HSC development itself (Elcheva *et al*, 2014). Using both lentiviral transduction and overexpression of modified mRNA, two distinct haemato‐endothelial programmes in differentiating hPSCs were demonstrated: pan‐myeloid (*ETV2* and *GATA2*) and erythro‐megakaryocytic (*GATA2* and *TAL1*). Both combinations induced human PSCs directly to HE but with differing capacities for further lineage specification. Hence, this suggests that the specification to discrete HPCs starts at the HE stage and is regulated by distinct transcriptional programmes. It is an interesting reflection that although complex genetic networks are undoubtedly involved in haematopoiesis, yet such a small subset of transcriptional regulators are capable of activating a TF network leading to HE formation with different functional capacities.

Multiple TFs were also used to convert lineage‐restricted progenitors derived from human PSCs (CD34^+^45^+^ myeloid precursors) to multipotential progenitors (Doulatov *et al*, 2013). Candidate TFs were initially selected based on those more highly expressed in HSCs compared to HPCs or mature cells in mouse & human gene expression data sets. An *in vitro* screen for self‐renewal capacity based on detecting clonogenic progenitors by serial plating was then employed. *HOXA9, ERG* and *RORA* were identified as conferring self‐renewal capacity when overexpressed *in vitro*. Using an inducible lentiviral system, ectopic expression of two additional factors, *SOX4* and *MYB*, conferred short‐term engraftment of the myeloid and erythroid lineages *in vivo* and the erythroid precursors underwent haemoglobin switching *in vivo*. However, despite obtaining T lymphoid potential *in vitro*, no lymphoid engraftment could be demonstrated *in vivo* and the overall engraftment level waned with time.

## Future perspectives

Transcription factor programming is a relatively new approach to the production and expansion of HSC/HPCs and our experience in human cells is still in its infancy. While the studies described in this review have undoubtedly progressed the field, we are still some way from achieving large‐scale production of HSCs and functional mature blood cells that could be used safely in the clinic.

To date, fully functional HSCs capable of long‐term multilineage engraftment and secondary transplantation have only being achieved in the mouse using the *in vivo* microenvironment for selection of HSCs (Riddell *et al*, 2014). The lack of success in generating fully functional HSCs probably reflects a failure to precisely recapitulate the *in vivo* developmental process. Mammalian embryonic haematopoiesis occurs in three waves within discrete anatomical niches (Yoder, 2014) and it is proposed that the ‘immature’ nature of human PSC‐derived cells (e.g. production of nucleated RBCs with fetal rather than adult globin) may be due to them arising from the first or second wave of haematopoiesis rather than the third ‘definitive’ wave. Current knowledge of human embryonic haematopoiesis is largely extrapolated from animal models and until recently we have lacked a clear understanding of a stepwise route to HSC production *in vivo*. The recent identification of surface markers of HSCs and their precursors (Rybtsov *et al*, 2011, 2014; Ivanovs *et al*, 2014) will provide vital reference points for their future production *in vitro*. The exact orchestration of both intrinsic and extrinsic signals mimicking the complex spatial, temporal and mechanical environment of the human embryo must be present if we are to produce HSCs *in vitro*.

Development of reprogramming technologies in well‐defined serum‐free, good manufacturing practice‐compliant human haematopoietic differentiation systems is essential if they are to be readily transferable to the clinic. Many of the differentiations systems used in studies to date have included serum or xenobiotic feeder cells that could not be directly translated into a therapeutic protocol.

In addition, many of the TFs used in the studies reviewed here are proto‐oncogenes and it is very likely that successful programming to HSCs will involve alteration of genetic networks common to both stem and cancer cells. This clearly raises important safety considerations as we move closer to clinical applications. Overexpression of a single TF is less likely to lead to malignant transformation as a ‘single hit’, without other co‐existent mutations. Yet the studies described suggest that overexpression of multiple TFs is likely to be more successful.

Thus the use of inducible expression strategies will be vital for ensuring only transient overexpression of TFs and to limit the oncogenic transformation of the cells produced. Most of the studies discussed have predominantly employed lentiviruses, which are randomly integrated into the genome and therefore may disrupt genome integrity and/or enhance oncogenic potential.

In the studies described here TFs were selected based on their known role in haematopoiesis or using a variety of screening strategies (Table 1) and have paved the way for further *in vitro* and *in vivo* screening for TF combinations to be tested. Future screening studies will benefit from using more sophisticated genome editing strategies such as the integration of transgenes into the AAVS locus using the CRISPR/CAS9 system to ensure robust and consistent expression (Sadelain *et al*, 2012). This locus provides a ‘safe harbour’, which avoids insertional mutagenesis that can occur using a random integration system. Ultimately, non‐integrative strategies that result in transient protein expression such as synthetic modified mRNA, non‐integrating plasmids (Bernal, 2013) and cell‐permeable TFs, as discussed earlier, could be safer alternatives.

Exciting advances in the production of synthetic TFs such as zinc finger, TALE and CAS9 proteins are likely to provide even more sophisticated tools to modulate the expression of endogenous genes that could bypass the need for transgene insertion altogether (Hockemeyer *et al*, 2011; Miller *et al*, 2011). For example, variant CAS9 proteins have been developed that can be directed to the regulatory regions of gene promoters using complementary guide RNAs and, when tethered to transcriptional activators (e.g VP64), they are able to activate the expression of that target gene (Gilbert *et al*, 2014; Sander & Joung, 2014). Very recently this strategy has been used to induce neuronal differentiation of human iPSCs via the activation of *NEUROG2* and *NEUROD1* (Chavez *et al*, 2015) and to improve directed differentiation to pancreatic progenitor‐like cells by activating simultaneously *SOX17, FOXA2, PDX1* and *NKX6‐1* (Balboa *et al*, 2015). This may well be an effective and safer strategy for forward programming PSCs into blood cell lineages.

The use of new programming technologies in combination with a better understanding of the inductive environment and the precise phenotype of the desired cell type may well lead to new breakthroughs during the next decade and bring us closer to the overall goal of the *in vitro* production of fully functional HSCs for use in the clinic.

## Author contributions

Jennifer Easterbrook: Conception & design, manuscript writing. Antonella Fidanza: Manuscript writing. Lesley M Forrester: Conception & design, manuscript writing.
